# Cost-Consequence Analysis of Advanced Imaging in Acute Ischemic Stroke Care

**DOI:** 10.3389/fneur.2021.774657

**Published:** 2021-11-26

**Authors:** Artem T. Boltyenkov, Gabriela Martinez, Ankur Pandya, Jeffrey M. Katz, Jason J. Wang, Jason J. Naidich, Elizabeth Rula, Pina C. Sanelli

**Affiliations:** ^1^Center for Health Innovations and Outcomes Research, Feinstein Institute for Medical Research, Manhasset, NY, United States; ^2^Siemens Healthcare, Malvern, PA, United States; ^3^Department of Radiology, Donald and Barbara Zucker School of Medicine at Hofstra-Northwell, Hempstead, NY, United States; ^4^Department of Health Policy and Management, School of Public Health, Harvard University, Boston, MA, United States; ^5^Department of Neurology, Donald and Barbara Zucker School of Medicine at Hofstra-Northwell, Hempstead, NY, United States; ^6^Harvey L. Neiman Health Policy Institute, Reston, VA, United States

**Keywords:** cost-consequence analysis, acute ischemic stroke, computerized tomography (CT), angiography, perfusion

## Abstract

**Introduction:** The purpose of this study was to illustrate the potential costs and health consequences of implementing advanced CT angiography and perfusion (CTAP) as the initial imaging in patients presenting with acute ischemic stroke (AIS) symptoms at a comprehensive stroke center (CSC).

**Methods:** A decision-simulation model based on the American Heart Association's recommendations for AIS care pathways was developed to assess imaging strategies for a 5-year period from the institutional perspective. The following strategies were compared: (1) advanced CTAP imaging: NCCT + CTA + CT perfusion at the time of presentation; (2) standard-of-care: non-contrast CT (NCCT) at the time of presentation, with CT angiography (CTA) ± CT perfusion only in select patients (initial imaging to exclude hemorrhage and extensive ischemia) for mechanical thrombectomy (MT) evaluation. Model parameters were defined with evidence-based data. Cost-consequence and sensitivity analyses were performed. The modified Rankin Scale (mRS) at 90 days was used as the outcome measure.

**Results:** The decision-simulation modeling revealed that adoption of the advanced CTAP imaging increased per-patient imaging costs by 1.19% ($9.28/$779.72), increased per-patient treatment costs by 33.25% ($729.96/$2,195.24), and decreased other per-patient acute care costs by 0.7% (–$114.12/$16,285.85). The large increase in treatment costs was caused by higher proportion of patients being treated. However, improved outcomes lowered the other per-patient acute care costs. Over the five-year period, advanced CTAP imaging led to 1.63% (66/4,040) more patients with good outcomes (90-day mRS 0-2), 2.23% (66/2,960) fewer patients with poor outcomes (90-day mRS 3-5), and no change in mortality (90-day mRS 6). Our CT equipment utilization analysis showed that the demand for CT equipment in terms of scanner time (minutes) was 24% lower in the advanced CTAP imaging strategy compared to the standard-of-care strategy. The number of EVT procedures performed at the CSC may increase by 50%.

**Conclusions:** Our study reveals that adoption of advanced CTAP imaging at presentation increases the demand for treatment of acute ischemic stroke patients as more patients are diagnosed within the treatment time window compared to standard-of-care imaging. Advanced imaging also leads to more patients with good functional outcomes and fewer patients with dependent functional status.

## Introduction

Stroke is one of the leading causes of morbidity and mortality in the United States. Imaging has been reported as the second largest and the fastest growing component of stroke care costs (1). The increased utilization of advanced imaging, such as angiography and perfusion using CT (CTAP) or MRI (MRAP), has been implicated as a contributing factor in the rising trend in stroke imaging costs (1).

Current guidelines endorsed by the American Heart Association (AHA) (2) state that in most patients, non-contrast CT (NCCT) imaging may be enough to obtain the necessary information for immediate stroke triage decisions. The guidelines emphasize that utilization of advanced imaging with angiography and perfusion should not delay treatment. The current standard-of-care practice is to perform NCCT at the time of initial presentation to determine if the patient is eligible for intravenous-thrombolytic therapy (IV-tPA). Advanced imaging such as CTAP or MRAP are utilized in patients who are otherwise eligible for endovascular therapy (EVT) (3, 4). With additional information from angiography and perfusion imaging, particularly regarding large vessel occlusion and the extent of brain infarction vs. salvageable brain tissue, patients may be better triaged for treatment with IV-tPA (3–5) and/or EVT at the time of initial presentation (6–8). Numerous studies have demonstrated that faster time-to-treatment from the acute stroke onset is associated with better clinical outcomes and functional independence (9–15). However, this relationship is non-linear. Therefore, even small efficiency improvements in the pre-treatment pathway, like the immediate performance of advanced imaging upon patient arrival to the emergency department (ED), may have a significant impact on the clinical outcomes of acute stroke patients. This is especially true for those with large vessel occlusion, who without treatment, or with delayed treatment, have the highest morbidity and mortality (16). Thus, some healthcare institutions have started to perform CTAP as the initial imaging strategy in all patients suspected of acute ischemic stroke at presentation to prevent delays in treatment (8).

Advanced CTAP imaging in acute ischemic stroke patients was shown to be cost-effective in prior work (17). In that study, the cost-effectiveness analysis was performed from a health care perspective. Institutions considering whether to adopt advanced CTAP imaging need to understand the costs and health consequences of this decision for their institution. In this research we look at the adoption of the advanced CTAP imaging from the institutional perspective, while using the many of the input parameters, assumptions and conclusions from the prior cost-effectiveness analysis (17).

The purpose of this study was to investigate the potential cost and health consequences of implementing CTAP at the time of initial presentation in the workflow of suspected acute ischemic stroke (AIS) patients, excluding stroke mimics, presenting to a comprehensive stroke center (CSC) within 24 h from symptom onset time (SOT) with National Institutes of Health Stroke Scale (NIHSS) score higher than or equal to 6, compared to the standard-of-care imaging strategy using advanced imaging only in select patients who may be eligible for EVT and return to the scanner for the additional imaging.

## Methods

**Figure 1:**
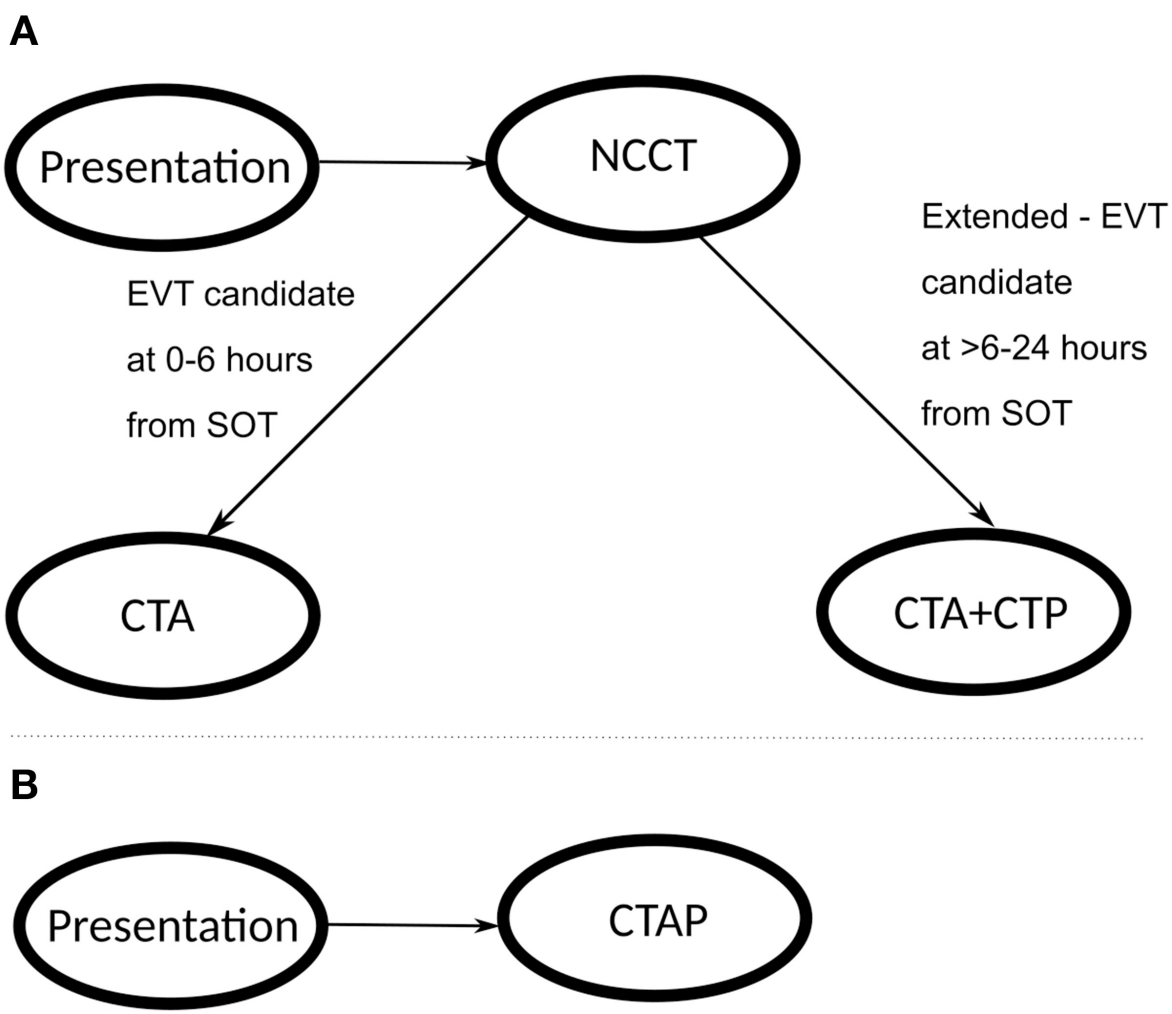
Patient workflow through the stroke imaging pathway; **(A)** represents the standard-of-care pathway, and **(B)** represents the advanced CTAP imaging pathway.

Institution review board (IRB) approval was not required because individual-level patient data was not utilized in this study. We developed a decision-simulation model of the acute stroke care pathways (17) from the perspective of a CSC using Microsoft Excel for Office 365 on Windows 10 operating system. The decision-simulation model algorithm for the patient work-up and clinical decision-making was based on the American Heart Association (AHA) Class-I recommendations for stroke management (2, 18). The structure of the patient workflow for the imaging strategies included: (1) Standard-of-care: all patients receive NCCT at the time of presentation; those patients who are eligible candidates for EVT within 0–6 h from SOT only receive CTA; those patients who are eligible candidates for Extended-EVT presenting at >6–24 h from SOT receive CTA+CTP; and (2) Advanced imaging: all patients receive CTAP (NCCT+CTA+CTP) at the time of presentation. In the advanced imaging strategy, we assume that perfusion imaging is performed on all patients within 24 h from SOT. Figure 1 describes the primary logic employed at the key decision points in the workflow algorithm. In this study, we focused on the time period from patient arrival to the time of treatment in the analysis. When no treatment is indicated, the time period terminated at the last imaging test in the ED. Further details of the patient workflow after these time-points are beyond the scope of this study.

The inclusion and exclusion criteria for suspected AIS patients in the model are consistent with the American Heart Association guidelines (2019 Update) for patient selection (2). Patients presenting with hemorrhagic stroke, stroke mimics, initial NIHSS score <6, and SOT over 24 h were excluded from the analysis, similar to the clinical trials (6, 8, 19–22). Detailed inclusion and exclusion criteria are listed in the Supplementary Table 3. We did not model all the potential scenarios in clinical practice in order to focus the analyses on the costs and health consequences of advanced CTAP imaging in acute ischemic stroke patients, which was shown to be cost-effective in prior work (17). The number of AIS patients admitted to our institution in prior years was utilized to extrapolate the trend in a linear model to estimate the number of stroke patients in the model in the next 5 years. Table 1 describes the total AIS patients over the historic 5 years at our institution and future 5 years calculated as a linear trend using the method of least squares.

**Table 1:** Total number of AIS patients modeled in the study over the 5-year period.

**Year**	**Number of patients**
Historic year #−5	1,188
Historic year #−4	1,131
Historic year #−3	1,328
Historic year #−2	1,329
Historic year #−1	1,455
Future year #1	1,506
Future year #2	1,613
Future year #3	1,670
Future year #4	1,766
Future year #5	1,838

*The volume of AIS patients in Future year #1 through Future year #5 was estimated as a linear projection of the trend based on the number of AIS patients admitted to our institution in the past 5 years using the method of least squares*.

Because our model focused on the CSC perspective, we limited the analysis to the institutional acute care costs within first 90-days after stroke, including imaging and treatment costs associated with these patients. We explored the time horizon of 5 years in this analysis (23). We modeled the cost and health effects impact on the dynamic cohort of patients for the period of 5 years, where continuously new patients with stroke were added to the cohort, and after 90 days post discharge were removed from the cohort. The annual acute care costs were calculated for both strategies during the 5-year period. We also calculated the per-patient acute care costs of each strategy and reported the total annual costs for a CSC to implement advanced CTAP imaging by multiplying the per-patient costs by the total number of patients each year. In addition, we incorporated the 90-day (within the first 90 days of stroke onset) and lifetime (over the remaining lifetime of the patient) cohort quality-adjusted life-years (QALY) for each strategy, and the percentage of patients in different 90-day modified Rankin scale (mRS) groups, representing functional independence (90-day mRS 0-2), functional dependence (90-day mRS 3-5), and death (90-day mRS 6). We measured 90-day and lifetime health impact using QALYs, a commonly used metric that combines the length and quality of life into a single value (24). The model input parameters were based on published literature as shown in Table 2, representing the baseline scenario.

**Table 2:** Cost data with minimum and maximum values adjusted for inflation to reflect values in 2019 U.S. dollars.

**Model Parameter**	**Baseline value (minimum-maximum)**	**References**
**Costs**		
IV-tPA	$7,518 ($7,217–$9,022)	(25, 26)
EVT	$15,714 ($15,085–$18,857)	(25, 26)
NCCT	$202 ($194–$242)	(1, 26, 27)
CTA ± CTP	$789 ($757–$947)	(1, 26, 27)
**Acute-care cost**		
Acute care costs within first 90 days after stroke (excluding imaging, IVT and EVT) mRS 0-2	$11,544 ($11,083–$13,853)	(26, 28, 29)
Acute care costs within first 90 days after stroke (excluding imaging, IVT and EVT) mRS 3-5	$26,818 ($25,745–$32,181)	(26, 28, 29)
Acute care costs within first 90 days after stroke (excluding imaging, IVT and EVT) mRS 6	$7,681 ($7,374–$9,217)	(26, 28, 29)
**90-day utilities**		
mRS 0-2	0.89	(30, 31)
mRS 3-5	0.33	(30, 31)
**Lifetime discounted QALYs**		
mRS 0-2	12.89	(32)
mRS 3-5	5.5	(32)
**Time of diagnostic tests, minutes**		
NCCT	5.00	(3)
CTA	15.00	(3)
CTA+CTP	25.00	(18)
CTAP (NCCT+CTA+CTP)	25.00	(18)
Interval between patients	10.00	(18)
**Probabilities of transitioning to 90-day modified Rankin scale (mRS): standard-of-care imaging**		
mRS 0-2	48.1340248%	(17)
mRS 3-5	35.2481406%	(17)
mRS 6	16.6178346%	(17)
**Probabilities of transitioning to 90-day modified Rankin scale (mRS): advanced imaging**		
mRS 0-2	48.9114442%	(17)
mRS 3-5	34.4948803%	(17)
mRS 6	16.5936755%	(17)

*Utilities values are average utility values for the group of patients with the corresponding 90-day mRS scores. Lifetime discounted QALYs are the expected quality adjusted life years of people having respective 90-day mRS scores. Time to perform each of the imaging procedure, and interval time between patients in the CT scanner*.

In this analysis, the initiation costs were set to $0 because we assumed that CSCs already had the necessary CT scanners and/or angiography equipment available for stroke patients both in standard-of-care and CTAP strategies. Thus, we focused this analysis on incremental costs associated with implementation of CTAP imaging for stroke care.

The ongoing operational and clinical acute care costs were derived from the published literature as shown in Table 2, which utilize Medicare CPT codes to estimate the costs. It is standard practice in health economics evaluations to use Medicare reimbursement as a substitute for actual costs (25, 33, 34) to minimize bias from practice variation. These costs include depreciation on all depreciable type assets that are used to provide covered services to beneficiaries (35). Total and per-patient costs were generated for the three main categories of interest: imaging, treatment, and other 90-day acute care costs. Other 90-day acute care costs consisted of the cost of hospital bed occupancy and the length of stay (28, 29). Sensitivity analyses were performed to calculate costs based on the variation of the input parameters in the model. By performing univariate sensitivity analyses, we determined the range from the least to the greatest per-patient costs. Table 2 shows the baseline, minimum and maximum cost values used in the sensitivity analyses. The input parameters for sensitivity analyses were based on published literature.

In order to assess the impact of a new imaging strategy on the utilization of the CT equipment, we performed a thorough literature review and identified the scanner time required for NCCT, CTA, and CTP imaging, as well as the time interval between patients, shown in Table 2. Scanner time refers to the amount of time that a CT scanner is occupied for a certain procedure. The procedures for which the scanner time was calculated were: NCCT, CTA, CTA+CTP, CTAP, and the interval time between patients.

The costs and outcomes for each imaging strategy were calculated separately. Then the costs and outcomes of the standard-of-care imaging scenario were separately subtracted from the costs and outcomes of the CTAP imaging scenario. The resulting differences in costs and outcomes represent the incremental differences reported in this analysis.

## Results

Adoption of the advanced CTAP imaging strategy increased per-patient imaging costs by 1.19% ($9.28/$779.72), increased per-patient treatment costs by 33.25% ($729.96/$2,195.24), and decreased per-patient other acute care costs by 0.7% (–$114.12/$16,285.85). The large increase in treatment costs was due to the higher proportion of patients being treated. Lower per-patient other acute care costs were mainly due to better health outcomes and shorter hospital stay.

The per-patient cost analysis for the two imaging strategies is shown in Table 3. While performing advanced CTAP imaging on the identical cohort of stroke patients, as in the standard-of-care strategy, the incremental imaging costs were higher by $9.28 per patient in the advanced CTAP imaging strategy. Since the costs were based on the Medicare CPT codes, the incremental costs of $9.28 in the advanced CTAP imaging strategy translates to the CSC receiving $9.28 more reimbursement revenue per patient. This higher cost was driven by greater utilization of the higher reimbursed CTAP imaging.

**Table 3:** Per-patient costs projection for the 5-year period for the standard-of-care and advanced CTAP imaging strategies.

	**Year 1**	**Year 2**	**Year 3**	**Year 4**	**Year 5**	**Average**	**Change**
**Annual per-patient costs**							
**Strategy 1: standard-of-care imaging**							
CTA	$439.11	$439.11	$439.11	$439.11	$439.11	$439.11	
CTA+CTP	$138.61	$138.61	$138.61	$138.61	$138.61	$138.61	
NCCT	$202.00	$202.00	$202.00	$202.00	$202.00	$202.00	
IV-tPA	$906.69	$906.69	$906.69	$906.69	$906.69	$906.69	
EVT	$1,288.55	$1,288.55	$1,288.55	$1,288.55	$1,288.55	$1,288.55	
Imaging costs	$779.72	$779.72	$779.72	$779.72	$779.72	$779.72	
Treatment costs	$2,195.24	$2,195.24	$2,195.24	$2,195.24	$2,195.24	$2,195.24	
Other acute care costs within first 90 days after stroke (excluding imaging, IVT and EVT)	$16,285.85	$16,285.85	$16,285.85	$16,285.85	$16,285.85	$16,285.85	
Total costs	$19,260.82	$19,260.82	$19,260.82	$19,260.82	$19,260.82	$19,260.82	
**Strategy 2: advanced imaging**							
CTA	$0.00	$0.00	$0.00	$0.00	$0.00	$0.00	
NCCT+CTA+CTP	$789.00	$789.00	$789.00	$789.00	$789.00	$789.00	
NCCT	$0.00	$0.00	$0.00	$0.00	$0.00	$0.00	
IV-tPA	$992.38	$992.38	$992.38	$992.38	$992.38	$992.38	
EVT	$1,932.82	$1,932.82	$1,932.82	$1,932.82	$1,932.82	$1,932.82	
Imaging costs	$789.00	$789.00	$789.00	$789.00	$789.00	$789.00	
Treatment costs	$2,925.20	$2,925.20	$2,925.20	$2,925.20	$2,925.20	$2,925.20	
Other acute care costs within first 90 days after stroke (excluding imaging, IVT and EVT)	$16,171.73	$16,171.73	$16,171.73	$16,171.73	$16,171.73	$16,171.73	
Total costs	$19,885.93	$19,885.93	$19,885.93	$19,885.93	$19,885.93	$19,885.93	
**Incremental costs**							
Imaging incremental costs	$9.28	$9.28	$9.28	$9.28	$9.28	$9.28	1.19%
Treatment incremental costs	$729.96	$729.96	$729.96	$729.96	$729.96	$729.96	33.25%
Other acute care 90-days incremental costs	-$114.12	-$114.12	-$114.12	-$114.12	-$114.12	-$114.12	−0.70%
Total incremental costs	$625.11	$625.11	$625.11	$625.11	$625.11	$625.11	3.25%

*Incremental costs section shows the per-patient difference between both strategies, where the corresponding value from the standard-of-care strategy is subtracted from the advanced CTAP imaging strategy*.

Our CT equipment utilization analysis showed that the demand for CT equipment in terms of scanner time (minutes) was 24% lower in the advanced CTAP imaging strategy compared to the standard-of-care strategy. Although executing the imaging protocols for NCCT, CTA and CTP in one session takes longer than performing only NCCT or CTA on an individual patient, 73.2% of the patients in the standard-of-care strategy return to the scanner for additional imaging with angiography (and some also with perfusion) if they are potentially eligible for EVT. Besides the penalty of the time spent on subsequent imaging for the same patient, additional burden is the time spent between imaging tests, as some time is needed for the first patient to leave the CT scanner, and the next one to arrive to the scanner.

Univariate sensitivity analyses revealed that the change for imaging costs in the 1st year ranged from –$46,264 to $77,676, the growth for treatment costs ranged from $1,060,335 to $1,293,210, the decline in other 90-day acute care costs ranged from –$232,672 to –$144,811. The largest variation was seen in the treatment costs; the imaging and other 90-day costs had a narrower variation between the lower and upper boundaries of the parameter values. Overall, the sensitivity analysis suggested that after implementation of advanced CTAP imaging, imaging costs would either grow or decline, treatment costs would definitely grow, and other 90-day acute care costs would definitely decline.

The average overall incremental care costs of the advanced CTAP imaging strategy for a CSC with 1,679 annual strokes were $1,049,322. Of the total incremental acute care costs in the 1st year, only 1.48% ($15,571/$1,049,322) was attributed to the growth in imaging costs, 116.77% ($1,225,314/$1,049,322) to the growth in treatment costs, and −8.26% (–$191,563/$1,049,322) to the decline in other 90-day acute care costs. The results of the costs analysis for 5 consecutive years after implementation, further details on the univariate sensitivity analysis, and CT equipment utilization analysis are included in Supplementary Material.

An analysis on the projected impact of the advanced CTAP imaging strategy on the number of EVT procedures performed at the CSC found that EVT procedures may increase by 50% and IV-tPA procedures may increase by 9% each year. Table 4 details the projected increase in the number of EVT and IV-tPA procedures for both imaging strategies.

**Table 4:** Estimated number of EVT and IV-tPA procedures in the advanced CTAP imaging and standard-of-care strategies for the 5-year period.

**Year**	**Number of patients**	**Number of EVT procedures**				**Number of IV-tPA procedures**			
		**Number of EVTs in advanced imaging scenario**	**Number of EVTs in standard-of-care scenario**	**Increase in number of EVTs**	**Percentage increase in number of EVTs**	**Number of IV-tPAs in advanced imaging scenario**	**Number of IV-tPAs in standard-of-care scenario**	**Increase in number of IV-tPAs**	**Percentage increase in number of IV-tPAs**
1	1,506	185	123	62	50%	199	182	17	9%
2	1,613	198	132	66	50%	213	195	18	9%
3	1,670	205	137	68	50%	220	201	19	9%
4	1,766	217	145	72	50%	233	213	20	9%
5	1,838	226	151	75	50%	243	222	21	9%
Total	8,393	1,031	688	343	50%	1,108	1,013	95	9%

An additional important result of our analysis is that in the CTAP imaging strategy, health outcomes changed. The impact of CTAP imaging on health outcomes for the 5-year time period is shown in Table 5. We found that on average the lifetime quality-adjusted life years of a patient in the advanced CTAP imaging strategy improved by 0.03 QALYs, compared to the standard-of-care strategy. This corresponds to $20,837 per QALY gained. On average, the 90-day utility increased by 0.0044 QALYs because more patients were treated in the CTAP imaging strategy. In this acute stroke cohort, we found that the clinical outcomes in the CTAP imaging strategy improved, with 13 more patients having better functional outcomes, defined by the 90-day mRS 0-2, and 13 fewer patients having a dependent functional status (90-day mRS 3-5) compared to the standard-of-care strategy on average per year. This corresponds to $79,448 per functional dependence avoided in the 1st year. We found no change in mortality (90-day mRS 6).

**Table 5:** Health impact projection of the number of people in each health outcome group for the advanced CTAP imaging and standard-of-care strategies for the 5 year period.

	**Year 1**	**Year 2**	**Year 3**	**Year 4**	**Year 5**	**Total**	**Average**
**Annual health outcomes**							
**Strategy 1: standard-of-care imaging**							
Lifetime QALYs of the cohort	8,839	9,467	9,803	10,369	10,790	49,267	9,853
Short term QALYs of the cohort	820	878	910	962	1,001	4,572	914
mRS 0-2	725	776	804	850	885	4,040	808
mRS 3-5	531	569	589	623	648	2,960	592
Deaths	250	268	277	293	305	1,393	279
**Strategy 2: advanced imaging**							
Lifetime QALYs of the cohort	8,884	9,515	9,853	10,422	10,845	49,519	9,904
90-day QALYs of the cohort	827	886	917	970	1,009	4,609	922
mRS 0-2	737	789	817	864	899	4,106	821
mRS 3-5	519	556	576	609	634	2,894	579
Deaths	250	268	277	293	305	1,393	279
**Incremental health impact**							
Lifetime QALYs of the cohort	45	48	50	53	55	252	50
Lifetime utility per person	0.0300	0.0300	0.0300	0.0300	0.0300		0.0300
90-day QALYs of the cohort	7	7	7	8	8	37	7
90-day utility per person	0.0045	0.0045	0.0044	0.0044	0.0043		0.0044
mRS 0-2	12	13	13	14	14	66	13
mRS 3-5	−12	−13	−13	−14	−14	−66	−13
Deaths	0	0	0	0	0	0	0
**Relationship between costs and outcomes**							
Cost per functional dependence avoided	$78,441	$77,550	$80,303	$78,872	$82,073		$79,448
Cost per QALY gained	$20,837	$20,837	$20,837	$20,837	$20,837		$20,837

*The incremental health impact of switching from the standard-of-care to the advanced CTAP imaging strategy is also shown*.

Finally, the model projected that over 5 years in the advanced CTAP imaging strategy, out of 8,393 AIS patients, 66 more patients would have a good functional outcome (90-day mRS 0-2), and 66 fewer patients would have a dependent functional status (90-day mRS 3-5), with no change in mortality (90-day mRS 6).

## Discussion

The main finding from this analysis is that performing advanced imaging using CTAP for AIS patients at presentation increases overall costs for a CSC, with the greatest impact from treatment costs, over the 5-year period. Our analyses show that CTAP imaging in moderate-to-severe acute ischemic stroke care leads to more patients being eligible for treatment within the time window, and thus undergoing endovascular therapy, translating to improved health outcomes. To our knowledge, no studies have analyzed the incremental costs and benefits associated with implementing advanced CTAP imaging in the initial evaluation of patients presenting with moderate-to-severe acute ischemic stroke symptoms from the healthcare provider perspective.

While costs were higher, primarily due to more EVT performed, our study showed that the transition to advanced CTAP imaging strategy led to an increase in the number of patients with good functional clinical outcomes (90-day mRS 0-2), while the number of patients with moderate to severe disability decreased (90-day mRS 3-5). There was no change in mortality (90-day mRS 6). Importantly, this implies that the costs for long-term care will also be reduced. At $20,837/QALY, the advanced CTAP imaging strategy should be considered appropriate for adoption in clinical care when considering a threshold of $50,000/QALY, which is customarily used as a threshold in health policy (4, 5, 17, 25, 34).

Furthermore, our model results, based on Medicare CPT reimbursement, showed that for diagnostic imaging, the advanced CTAP imaging strategy is more costly than the standard-of-care strategy. However, the analysis of the scanner times showed that the advanced imaging strategy required less scanner time than the standard-of-care strategy, and therefore, the diagnostic imaging component could actually be less costly. We explain this discrepancy as the scanner time analysis represents opportunity costs for the hospital. With 24% less scanner utilization in the advanced CTAP imaging strategy, the remaining scanner time can potentially be used for other patients.

Although we showed that the demand for CT equipment in terms of minutes was lower in the advanced CTAP imaging strategy, the demand for angiography equipment to perform EVTs grew in the advanced imaging strategy. Having more stroke cases treated with EVT potentially indicates higher reimbursement revenue for the hospital. The potential effect of whether new angiography equipment needs to be purchased strongly depends on the case mix of the particular institution. Our analysis suggests that when contemplating shifting to the advanced CTAP imaging strategy in stroke care, an institution must consider the interventional neuroradiology capacity to handle the increased volume of EVT. If EVT capacity is insufficient, then implementation of the CTAP imaging strategy may lead to delayed treatment for EVT-eligible patients. This delay, in turn, could lead to worse outcome for AIS patients if treatment is not initiated in a timely manner.

The first limitation of this study is that we did not account for any initiation costs. Initiation costs depend on the availability of radiologists and/or CT scanners in a particular institution. At our institution ED CT scanners run 24/7 and they are currently not utilized at full capacity. We have hardware, software and personnel to accommodate peaks in the demand for radiology service and transition to advanced CTAP imaging. Therefore, we might not need any initiation costs going from the standard of care to advanced CTAP imaging. On the other hand, institutions currently running their CT scanners at full capacity might need an additional scanner for this transition to account for peaks in the demand. CSCs need to add our analysis to their baseline situation to determine if there are any relevant initiation costs for the transition at their institution.

Another limitation of our study is that we used Medicare CPT reimbursement as a surrogate for the costs to the institution. Although this approach may not accurately represent the actual costs at the specific institution level, it remains standard practice in health economics studies in radiology (25, 33, 34). Without more precise methodology available, we conformed to standard practice in this study with the potential for better generalizability to other institutions.

The next limitation of our study is that we did not measure discounted costs against an effectiveness outcome, as would be performed in a cost-effectiveness analysis from a broader societal perspective (17), which was out of the scope of this study. Healthcare institutions in the United States deciding on whether to implement advanced CTAP imaging for all incoming AIS patients might want or need to know the undiscounted costs and health impact of the proposed care pathway change in each of the next 1–5 years (23). Lifetime discounted costs and outcomes, which are the typical output of a cost-effectiveness analysis, are useful additional information, but shorter-term undiscounted costs and health impact information are often helpful complementary inputs for decision-making at the institutional level (23).

Furthermore, we do not have a reliable source of information how the costs used in our model will change, or how the treatment and care methods for AIS will change, in the next 5 years. We performed sensitivity analyses in our study in order to account for this uncertainty.

Besides, another limitation of our study is that we didn't have the reliable data to model MT outcomes of patients presenting in the LKWA >6–24-h time window. We used extrapolated data from our previously published population-based study (17) to model clinical outcomes of some stroke imaging subtypes because adequate data did not exist in the literature. Importantly, our model reflects stroke care pathways recommended by the AHA guidelines (2) with workflow from RCTs, which report arrival-to-treatment times between 74 and 148 min (9).

Finally, we excluded patients with NIHSS <6 from our model, similar to the clinical trials (6, 8, 19–22). A separate study is required to analyze the cost-consequence of advanced CTAP imaging of suspected AIS in patients presenting with NIHSS <6.

In this study we analyzed the incremental costs of advanced CTAP imaging for acute stroke care from the CSC perspective. Since actual costs are highly variable between different types of healthcare institutions, future research may analyze the adoption of advanced CTAP imaging strategy in practice settings other than a CSC.

## Conclusions

Advanced CTAP imaging in acute ischemic stroke care increases diagnostic and treatment costs with more patients eligible for treatment in the time window undergoing endovascular therapy, thus leading to improved stroke health outcomes. Consequently, CTAP imaging leads to more patients with good functional outcomes (90-day mRS 0-2), fewer patients with dependent functional status (90-day mRS 3-5) and unchanged mortality (90-day mRS 6). The present study can be used as a resource-planning tool for CSCs considering adoption of advanced CTAP imaging protocols for acute ischemic stroke patients.

## Data Availability Statement

The original contributions presented in the study are included in the article/Supplementary Material, further inquiries can be directed to the corresponding author/s.

## Author Contributions

All authors listed have made a substantial, direct, and intellectual contribution to the work and approved it for publication.

## Funding

Research reported in this publication was supported by Northwell Health, National Institute of Neurological Disorders and Stroke of the National Institutes of Health under award number R56NS114275, and Siemens Medical Solutions USA, Inc. The content is solely the responsibility of the authors and does not necessarily represent the official views of the National Institutes of Health.

## Author Disclaimer

The content is solely the responsibility of the authors and does not necessarily represent the official views of the National Institutes of Health.

## Conflict of Interest

This study received funding from Northwell Health, National Institute of Neurological Disorders and Stroke of the National Institutes of Health under award number R56NS114275, and Siemens Medical Solutions USA, Inc. The funder had the following involvement with the study: - Northwell Health: Involvement in study design, data collection and analysis, decision to publish, or preparation of the manuscript through its employees JK, JW, JN, and PS. - National Institute of Neurological Disorders and Stroke of the National Institutes of Health: The funder was not involved in the study design, collection, analysis, interpretation of data, the writing of this article or the decision to submit it for publication. - Siemens Medical Solutions USA, Inc.: Involvement in study design, data collection and analysis, decision to publish, or preparation of the manuscript through its employees AB and GM. Research reported in this publication was supported by Northwell Health, National Institute of Neurological Disorders and Stroke of the National Institutes of Health under award number R56NS114275, and Siemens Medical Solutions USA, Inc. All authors declare no other competing interests.

## Publisher's Note

All claims expressed in this article are solely those of the authors and do not necessarily represent those of their affiliated organizations, or those of the publisher, the editors and the reviewers. Any product that may be evaluated in this article, or claim that may be made by its manufacturer, is not guaranteed or endorsed by the publisher.
