# Supplementary material for: Cost-Consequence Analysis of Advanced Imaging in Acute Ischemic Stroke Care
Source: Front Neurol. 2021 Nov 26;12:774657. doi: 10.3389/fneur.2021.774657 (PMC8662622; doi:10.3389/fneur.2021.774657)
Supplement: Supplementary file 1 [file Data_Sheet_1.docx]

**Supplemental Materials**

**Sensitivity Analysis**

We performed sensitivity analyses by varying the cost input baseline parameters in the range from minimum to maximum value. The resulting sensitivity analysis charts are shown in **Figure 1S**, **Figure 2S** and **Figure 3S** below.

**Calculation of the time impact.**

We performed the analysis of the time impact on CT scanner utilization by switching from the standard-of-care strategy to advanced CTAP imaging strategy in stroke care. **Table 1S** below illustrates the scanner time used in each of the imaging strategies, and the scanner time utilization difference between both strategies.

**Calculation of the cost impact on the acute stroke cohort.**

We performed the analysis of the cost impact on the acute ischemic stroke cohort of our institution for 5 years by switching from the standard-of-care strategy to advanced CTAP imaging strategy in stroke care. **Table 2S** below illustrates the cost impact.

**Supplemental Figures**

Figure 1S: Sensitivity analysis of the annual imaging incremental costs in the 5 year period. The chart shows the annual incremental imaging costs of switching from standard-of-care to advanced CTAP imaging strategy depending on the true value of NCCT and CTAP costs.

- *NCCT_min – minimal possible cost of NCCT imaging*
- *NCCT_max – maximal possible cost of NCCT imaging*
- *All default – incremental imaging costs if NCCT and CTAP costs are at baseline*
- *CTAP_min – minimal possible cost of CTAP imaging*
- *CTAP_max – maximal possible cost of CTAP imaging*

Figure 2S: Sensitivity analysis of the annual treatment incremental costs. The chart shows the annual incremental treatment costs of switching from standard-of-care to advanced CTAP imaging strategy depending on the true value of IV-tPA and EVT costs.

- *IVTPA_min – minimal possible cost of IVTPA treatment*
- *IVTPA_max – maximal possible cost of IVTPA treatment*
- *All default – incremental treatment costs if IVTPA and EVT costs are at baseline*
- *EVT_min – minimal possible cost of EVT treatment*
- *EVT_max – maximal possible cost of EVT treatment*

Figure 3S: Sensitivity analysis of the incremental annual 90-days other acute care costs after stroke (excluding imaging, IVT and EVT). The chart shows the annual other acute care 90-days after stroke incremental costs of switching from standard-of-care to advanced CTAP imaging strategy depending on the true value of IV-tPA and EVT costs.

- *mRS 0-2 min – minimal possible other acute care costs within first 90 days after stroke (excluding imaging IVT and EVT) for patients with 90 days mRS 0-2*
- *mRS 0-2 max – maximal possible other acute care costs within first 90 days after stroke (excluding imaging IVT and EVT) for patients with 90 days mRS 0-2*
- *All default – incremental other 90-days acute care costs after stroke (excluding imaging, IVT and EVT) at baseline*
- *mRS 3-5 min – minimal possible other acute care costs within first 90 days after stroke (excluding imaging IVT and EVT) for patients with 90 days mRS 3-5*
- *mRS 3-5 max – maximal possible other acute care costs within first 90 days after stroke (excluding imaging IVT and EVT) for patients with 90 days mRS 3-5*
- *mRS 6 min – minimal possible other acute care costs within first 90 days after stroke (excluding imaging IVT and EVT) for patients with 90 days mRS 6*
- *mRS 6 max – maximal possible other acute care costs within first 90 days after stroke (excluding imaging IVT and EVT) for patients with 90 days mRS 6*

| **Strategy 1: Standard-of-care imaging** | | | | | | | | | |
| --- | --- | --- | --- | --- | --- | --- | --- | --- | --- |
| **Year** | **CTA** | | **CTA+CTP or CTAP** | **NCCT** | | **Intervals** | | **Total** | |
| 1 | 25,141 | | 10,581 | 7,529 | | 26,084 | | 69,336 | |
| 2 | 26,927 | | 11,333 | 8,064 | | 27,936 | | 74,260 | |
| 3 | 27,883 | | 11,735 | 8,350 | | 28,928 | | 76,896 | |
| 4 | 29,493 | | 12,413 | 8,832 | | 30,598 | | 81,336 | |
| 5 | 30,690 | | 12,917 | 9,191 | | 31,840 | | 84,637 | |
| **Strategy 2: Advanced imaging** | | | | | | | | | |
| 1 | 0 | 37,645 | | | 0 | | 15,058 | | 52,703 |
| 2 | 0 | 40,319 | | | 0 | | 16,127 | | 56,446 |
| 3 | 0 | 41,750 | | | 0 | | 16,700 | | 58,450 |
| 4 | 0 | 44,161 | | | 0 | | 17,664 | | 61,825 |
| 5 | 0 | 45,953 | | | 0 | | 18,381 | | 64,334 |

Table 1S: Projection of the time impact (in minutes) for the advanced CTAP imaging and standard-of-care strategies for the 5 year period. For each of the two imaging strategies we calculate the total time needed to scan all the patients in each year. For the standard-of-care strategy all patients undergo NCCT imaging, and some CTA or CTA+CTP imaging. In the advanced imaging strategy, all patients undergo CTAP imaging. The last column “Total” show the total number of minutes needed to scan all patients, including the time interval between patients. We assume in this analysis that patients continuously arrive one after another.

|  | **Year 1** | **Year 2** | **Year 3** | **Year 4** | **Year 5** | **Average** | **Change** |
| --- | --- | --- | --- | --- | --- | --- | --- |
| **Annual Costs** | | | | | | | |
| **Strategy 1: Standard-of-care imaging** | | | | | | | |
| CTA only | $661,218 | $708,177 | $733,317 | $775,662 | $807,137 | $737,102 |  |
| CTA+CTP | $208,719 | $223,542 | $231,477 | $244,844 | $254,779 | $232,672 |  |
| NCCT only | $304,172 | $325,773 | $337,338 | $356,818 | $371,297 | $339,080 |  |
| IV-tPA | $1,365,299 | $1,462,261 | $1,514,171 | $1,601,605 | $1,666,596 | $1,521,986 |  |
| EVT | $1,940,296 | $2,078,093 | $2,151,865 | $2,276,122 | $2,368,484 | $2,162,972 |  |
| Imaging costs | $1,174,109 | $1,257,492 | $1,302,133 | $1,377,323 | $1,433,213 | $1,308,854 |  |
| Treatment costs | $3,305,595 | $3,540,354 | $3,666,036 | $3,877,726 | $4,035,080 | $3,684,958 |  |
| Other acute care costs within first 90 days after stroke (excluding imaging, IVT and EVT) | $24,523,239 | $26,264,848 | $27,197,246 | $28,767,717 | $29,935,075 | $27,337,625 |  |
| Total costs | $29,002,942 | $31,062,694 | $32,165,415 | $34,022,766 | $35,403,368 | $32,331,437 |  |
| **Strategy 2: Advanced imaging** | | | | | | | |
| CTA only | $0 | $0 | $0 | $0 | $0 | $0 |  |
| NCCT+CTA+CTP | $1,188,076 | $1,272,452 | $1,317,624 | $1,393,708 | $1,450,263 | $1,324,425 |  |
| NCCT only | $0 | $0 | $0 | $0 | $0 | $0 |  |
| IV-tPA | $1,494,320 | $1,600,444 | $1,657,260 | $1,752,956 | $1,824,089 | $1,665,814 |  |
| EVT | $2,910,443 | $3,117,139 | $3,227,797 | $3,414,182 | $3,552,726 | $3,244,458 |  |
| Imaging costs | $1,188,076 | $1,272,452 | $1,317,624 | $1,393,708 | $1,450,263 | $1,324,425 |  |
| Treatment costs | $4,404,763 | $4,717,584 | $4,885,057 | $5,167,139 | $5,376,815 | $4,910,272 |  |
| Other acute care costs within first 90 days after stroke (excluding imaging, IVT and EVT) | $24,351,398 | $26,080,803 | $27,006,667 | $28,566,133 | $29,725,311 | $27,146,062 |  |
| Total costs | $29,944,237 | $32,070,839 | $33,209,348 | $35,126,980 | $36,552,389 | $33,380,759 |  |
| **Incremental costs** | | | | | | | |
| Imaging incremental costs | $13,968 | $14,960 | $15,491 | $16,385 | $17,050 | $15,571 | 1.19% |
| Treatment incremental costs | $1,099,169 | $1,177,230 | $1,219,022 | $1,289,412 | $1,341,735 | $1,225,314 | 33.25% |
| Other acute care 90-days incremental costs | -$171,841 | -$184,045 | -$190,579 | -$201,584 | -$209,764 | -$191,563 | -0.70% |
| Total incremental costs | **$941,295** | **$1,008,144** | **$1,043,933** | **$1,104,214** | **$1,149,022** | **$1,049,322** | **3.25%** |

Table 2S: Five years costs projection for the standard-of-care and advanced CTAP imaging strategies. Incremental costs section shows the difference between both strategies, where the corresponding value from standard-of-care strategy is subtracted from the advanced CTAP imaging strategy.

| **Inclusion criteria** | **Exclusion criteria** |
| --- | --- |
| Patients presenting with anterior circulation acute ischemic stroke | Intracranial hemorrhage (ICH) identified by CT |
| Age: 18 through 82 years (i.e., candidates must have had their 18th birthday, but not had their 83rd birthday) | Pre-stroke mRS score of ≥ 2 (indicating previous disability) |
| Arterial occlusion on CT angiogram (CTA) of the ICA, M1 or M2 | Hypodensity in >1/3 MCA territory on non-contrast CT |
| Mismatch - Using CT perfusion imaging with a Tmax >6 second delay perfusion volume and CT-rCBF ischemic core volume  a) Mismatch ratio of greater than 1.2, and  b) Absolute mismatch volume of greater than 10 ml, and  c) Ischemic core lesion volume of less than 70mL | Contraindication to imaging with contrast agents |
| NIHSSS ≥ 6 at the time of presentation. | Any terminal illness such that the patient would not be expected to survive more than 1 year |
| Symptom Onset Time (SOT) ≤ 24 hours | Females of childbearing potential who are known to be pregnant and/or lactating or who have positive pregnancy tests on admission. |
|  | Previous stroke within last three months |
|  | Recent past history or clinical presentation of ICH, subarachnoid hemorrhage (SAH), arterio-venous (AV) malformation, aneurysm, or cerebral neoplasm |
|  | Current use of oral anticoagulants and a prolonged prothrombin time (INR > 1.7) |
|  | Use of heparin, except for low dose subcutaneous heparin, in the previous 48 hours and a prolonged activated partial thromboplastin time exceeding the upper limit of the local laboratory normal range |
|  | Use of glycoprotein IIb-IIIa inhibitors within the past 72 hours. Prior use of single or dual agent oral platelet inhibitors (clopidogrel and/or low-dose aspirin) permitted |
|  | Clinically significant hypoglycaemia |
|  | Uncontrolled hypertension defined by a blood pressure > 185 mmHg systolic or >110 mmHg diastolic on at least 2 separate occasions at least 10 minutes apart, or requiring aggressive treatment to reduce the blood pressure to within these limits |
|  | Hereditary or acquired hemorrhagic diathesis |
|  | Gastrointestinal or urinary bleeding within the preceding 21 days |
|  | Major surgery within the preceding 14 days |
|  | Presumed septic embolus, or suspicion of bacterial endocarditis |
|  | Presumed pericarditis including pericarditis after acute myocardial infarction |
|  | Suspicion of aortic dissection |
|  | Recent (within 30 days) trauma, with internal injuries or ulcerative wounds |
|  | Recent (within 90 days) severe head trauma or head trauma with loss of consciousness |
|  | Patients that require hemodialysis or peritoneal dialysis, or who have a contraindication to an angiogram for whatever reason |
|  | Patients with an arterial puncture at a non-compressible site or a lumbar puncture in the previous 7 days |
|  | Patients with a seizure at onset of stroke |
|  | Stroke mimics |

Table 3S: Detailed inclusion and exclusion criteria.
